# Smoking and secondhand smoke exposure and carotid intima-media thickness: Baseline data from the Aidai Cohort Study in Japan

**DOI:** 10.18332/tid/175632

**Published:** 2024-01-19

**Authors:** Makoto Saito, Yoshihiro Miyake, Keiko Tanaka, Chisato Nagata, Hidenori Senba, Yasuko Hasebe, Toyohisa Miyata, Takashi Higaki, Eizen Kimura, Bunzo Matsuura, Osamu Yamaguchi, Ryuichi Kawamoto

**Affiliations:** 1 Integrated Medical and Agricultural School of Public Health, Ehime University, Toon, Japan; 2 Department of Cardiology, Kitaishikai Hospital, Ozu, Japan; 3 Department of Epidemiology and Public Health, Ehime University Graduate School of Medicine, Toon, Japan; 4 Research Promotion Unit, Translation Research Center, Ehime University Hospital, Toon, Japan; 5 Center for Data Science, Ehime University, Matsuyama, Japan; 6 Department of Epidemiology and Preventive Medicine, Gifu University Graduate School of Medicine, Gifu, Japan; 7 Department of Internal Medicine, Matsuyama Shimin Hospital, Matsuyama, Japan; 8 Junpu Health Care Center, Toon, Japan; 9 Department of Regional Pediatrics and Perinatology, Ehime University Graduate School of Medicine, Toon, Japan; 10 Department of Medical Informatics, Ehime University Graduate School of Medicine, Toon, Japan; 11 Department of Lifestyle-Related Medicine and Endocrinology, Ehime University Graduate School of Medicine, Toon, Japan; 12 Department of Cardiology, Pulmonology, Hypertension and Nephrology, Ehime University Graduate School of Medicine, Toon, Japan; 13 Department of Community Medicine, Ehime University Graduate School of Medicine, Toon, Japan

**Keywords:** carotid intima-media thickness, cross-sectional study, Japanese, smoking, secondhand smoke exposure

## Abstract

**INTRODUCTION:**

Epidemiological evidence regarding the relationship between smoking and secondhand smoke (SHS) exposure and carotid intima-media thickness (CIMT) has been limited in Asian populations. Employing baseline data from the Aidai Cohort Study, Japan, we evaluated the evidence in this cross-sectional study.

**METHODS:**

Study subjects were 727 men aged 35–88 years and 1297 women aged 34–85 years. Information on smoking, SHS exposure, and confounders was obtained through a self-administered questionnaire. An automated carotid ultrasonography device was used to measure the right and left CIMT. The greatest CIMT measurement in the left or right common carotid artery was considered the maximum CIMT, and a maximum CIMT >1.0 mm was indicative of carotid wall thickening. Age, alcohol consumption, leisure time physical activity, hypertension, dyslipidemia, diabetes mellitus, body mass index, waist circumference, employment, and education level were adjusted at one time.

**RESULTS:**

The prevalence of carotid wall thickening was 13.0%. The prevalence of never smoking was 30.5% in men and 90.1% in women. Among those who had never smoked, the prevalence of never SHS exposure at home and work was 74.3% and 48.2% in men and 38.3% and 56.3% in women, respectively. Active smoking and pack-years of smoking were independently positively related to carotid wall thickening regardless of sex, although the association with current smoking in women was not significant. Independent positive relationships were shown between former smoking and pack-years of smoking and maximum CIMT in men but not in women. No significant relationships were found between SHS exposure at home and work and carotid wall thickening or maximum CIMT in either men or women.

**CONCLUSIONS:**

Active smoking, especially pack-years of smoking, was positively associated with carotid wall thickening in both sexes. Such positive associations with maximum CIMT were found only in men; however, interactions between smoking and sex were not significant.

## INTRODUCTION

Cigarette smoking is one of the most important modifiable risk factors for cardiovascular disease (CVD)^1–3^. Atherosclerosis is associated with the development of CVD and is also affected by smoking habits^2^. In addition, it has been suggested that secondhand smoke (SHS) exposure is related to the progression of atherosclerosis^4–8^.

There are various methods for estimating the status of atherosclerosis. Of these, carotid intima-media thickness (CIMT) is a useful marker for subclinical atherosclerosis because of its strong association with incident CVD and stroke events^9–12^. Many reports have been published regarding the consistent positive association between smoking and CIMT^2^. Although there is evidence of sex differences in the association between smoking and CIMT, the evidence in Asian populations is insufficient^13–15^. In addition, there has been a paucity of reports on the exposure–response relationship between smoking and CIMT and the association between SHS exposure and CIMT in adult Asian populations^7,16^. Here, we conducted a cross-sectional study to examine the associations of smoking and SHS exposure with carotid wall thickening and maximum CIMT in Japanese men and women using baseline data from the Aidai Cohort Study (AICOS) conducted in Yawatahama, Uchiko, Seiyo, and Ainan, which accounts for 0.089% of Japan’s total population.

## METHODS

### Study population

The AICOS is a currently ongoing prospective cohort study designed to clarify risk and protective factors for various health problems that was initiated in 2015^17,18^. The baseline AICOS survey was conducted in Yawatahama City in 2015, Uchiko Town in 2016, and in Seiyo City and Ainan Town in 2017, with total approximate populations of 36000, 17000, 38000, and 22000, respectively. These municipalities are among 20 municipalities in Ehime Prefecture on Shikoku Island, located south of the main island of Japan. The baseline study enrolled 798, 347, 524, and 755 participants in Yawatahama City, Uchiko Town, Seiyo City, and Ainan Town, respectively, and the participants were recruited from individuals who had undergone health checkups conducted by their municipality of residence or through alternative recruitment procedures. A total of 2424 participants, aged 33–89 years (including 894 men aged 35–89 years and 1530 women aged 33–85 years), completed the baseline questionnaire after providing written informed consent. For the present analyses, a total of 2024 study participants (727 men aged 35–88 years and 1297 women aged 34–85 years) were included after excluding 258 participants who had missing or questionable data for the study variables and 142 participants who self-reported a history of cardiovascular disease. The Ehime University Graduate School of Medicine ethics committee approved the AICOS study.

### Measurements

An automatic on-screen carotid ultrasound system (GM - 72P00A , The CardioHealth Station; Panasonic Healthcare Co., Ltd., Ehime, Japan) was used by one of two trained laboratory technicians and two medical doctors to measure left and right CIMT of the common carotid artery within a specified time limit. The UK Biobank reported high face validity and reproducibility of this automated device^19^. Moreover, it has been validated for accurate measurements even by novice operators^20^. Participants were placed in a supine position on a 180° examination bed with their head rotated against a gauge angled to 45°. All images were taken from the far wall of the distal common carotid artery, 1 cm proximally from the bifurcation. When the region of interest was correctly identified and software image criteria were met, an image was automatically frozen at end-diastole, and CIMT was calculated over a 10 mm length using automated boundary detection. The maximum CIMT was determined as the greatest CIMT observed in the left or right common carotid artery. According to the guidelines of the Japan Academy of Neurosonology and the Japan Society of Ultrasonics in Medicine, carotid wall thickening is defined as a maximum CIMT greater than 1.0 mm^21,22^.

A self-administered questionnaire was used to collect data on age, sex, smoking status, SHS exposure at home and at work, alcohol consumption, physical activity, medication use for hypertension, hypercholesterolemia, and diabetes, employment, and education. Research technicians conducted phone interviews to clarify any missing or implausible responses. Never smoking was defined as having smoked <100 cigarettes over one’s lifetime. Former smoking was defined as having smoked ≥100 cigarettes over one’s lifetime, but having quit smoking by the time of the survey. Current smoking was defined as having smoked ≥100 cigarettes over one’s lifetime and still smoking at the time of the survey. Cumulative exposure to cigarette smoking (pack-years of smoking) was calculated by multiplying the average number of packs smoked per day (cigarettes smoked per day/20) by the number of years smoked, regardless of whether smoking status was former or current. SHS exposure at home and at work was assessed, respectively, by the following questions: ‘Have you ever lived in the same house with a regular smoker for not less than one year?’ and ‘Have you ever worked in the same office with a regular smoker for not less than one year?’. Pack-years of SHS exposure at home were estimated by multiplying the average number of packs exposed to SHS at home per day by the number of years exposed to SHS at home. Leisure time physical activity was determined based on whether the participants engaged in any moderate to vigorous physical activity; for example, playing golf, walking briskly, jogging, gardening, or playing tennis for at least 30 min once a week. After the participant had rested for at least 5 min in a seated position, blood pressure measurements were recorded twice using an automated sphygmomanometer, and the latter value was used in this study. The presence of hypertension was determined based on systolic blood pressure ≥140 mmHg, diastolic blood pressure ≥90 mmHg, or current use of antihypertensive medication^23^. Following an overnight fast, blood was drawn from the antecubital vein, and serum levels of low-density and high-density lipoprotein cholesterol, triglycerides, plasma glucose, and hemoglobin A1c were determined by a third-party laboratory (Shikoku Chuken, Ehime, Japan). Dyslipidemia was diagnosed when serum low-density lipoprotein cholesterol levels were ≥140 mg/dL, high-density lipoprotein cholesterol levels were below 40 mg/dL, triglyceride levels were ≥150 mg/dL, or the participant was currently taking medication to lower cholesterol^24^. Diabetes mellitus was diagnosed when a fasting plasma glucose level was ≥126 mg/dL, hemoglobin A1c levels were ≥6.5%, or the participant was currently taking medication for diabetes^25^. Body height and weight were recorded while the study participant was wearing light clothing and without shoes, and body mass index (BMI, kg/m^2^) was calculated. Waist circumference was measured at the level of the navel with the participant standing.

### Statistical analysis

Smoking status was classified into three categories (never, former, and current), pack-years of smoking into three categories (none, 0.05 to <30, and ≥30 in men and none, 0.015 to <10, and ≥10 in women), SHS exposure at home into three categories (never, former, and current), pack-years of SHS exposure at home into three categories (none, 0 to <10, and ≥10 in men and none, 0 to <15, and ≥15 in women), SHS exposure at work into three categories (never, former, and current), and years of SHS exposure at work into three categories (none, 1 to <23, and ≥23 in men and none, 1 to <11, and ≥11 in women). Pack-years of smoking among former or current smokers, pack-years of SHS exposure at home among those who had been exposed to SHS at home, and years of SHS exposure at work among those who had been exposed to SHS at work were classified at approximately the median point with the sexes separated.

*A priori*, possible confounding factors examined in this study were age, alcohol consumption, leisure time physical activity, hypertension, dyslipidemia, diabetes mellitus, BMI, waist circumference, employment, and education level. Age, BMI, and waist circumference were continuous variables. Adjusted odds ratios (ORs) and 95% confidence intervals (CIs) for the relationships between active smoking or SHS exposure and carotid wall thickening, were estimated using multiple logistic regression analysis. Analysis of covariance was used to calculate the adjusted mean of the maximum CIMT according to active smoking or SHS exposure, with adjustment for confounding factors. A trend in an association was assessed using a multiple logistic regression model or multiple linear regression analysis, assigning consecutive integers to the categories of the exposure variables. A two-sided p-value of less than 0.05 was regarded as statistically significant. Since the maximum CIMT distribution was skewed to the right, the natural logarithms of the values were used. Thus, the mean maximum CIMT values and their 95% CIs were expressed as geometric means. All statistical analyses were carried out utilizing SAS software version 9.4 (SAS Institute, Inc., Cary, NC, USA).

## RESULTS

The maximum CIMT range of the 2024 study participants varied from 0.423 to 1.925 mm. The median (95th percentile) value was 0.770 mm (1.116 mm). The geometric mean (95% CI) of the maximum CIMT was 0.783 mm (0.775–0.790), and 13.0% of the study population had carotid wall thickening. In men, pack-years of smoking was positively associated with age, hypertension, and dyslipidemia, and inversely associated with employment; in women, pack-years of smoking was positively associated with alcohol consumption and employment, and inversely related to age (Table 1).

**Table 1:** Characteristics of study participants according to pack-years of smoking, baseline data from the Aidai Cohort Study, Japan, 2015–2017 (N=2024)

*Characteristics*	*Men*					*Women*				
	*Overall* *n (%)*	*Pack-years of smoking*				*Overall*	*Pack-years of smoking*			
		*None* *n (%)*	*0.05 to < 30* *n (%)*	*≥30* *n (%)*	*p for trend^a^*		*None* *n (%)*	*0.015 to <10* *n (%)*	*≥10* *n (%)*	*p for trend^a^*
**Total**, n	727	222	272	233		1297	1168	66	63	
**Age** (years), median (IQR)	65.0 (57.0–70.0)	65.0 (55.0–71.0)	64.0 (53.0–69.0)	67.0 (62.0–70.0)	0.005	63.0 (55.0–69.0)	64.0 (56.0–69.0)	58.0 (46.0–63.0)	58.0 (50.0–66.0)	<0.0001
**Alcohol consumption**					0.48					<0.0001
Never	117 (16.1)	42 (18.9)	39 (14.3)	36 (15.5)		752 (58.0)	711 (60.8)	19 (28.8)	22 (34.9)	
Former	55 (7.6)	17 (7.7)	16 (5.9)	22 (9.4)		74 (5.7)	57 (4.9)	11 (16.7)	6 (9.5)	
Current	555 (76.3)	163 (73.4)	217 (79.8)	175 (75.1)		471 (36.3)	400 (34.3)	36 (54.6)	35 (55.6)	
**Leisure time physical activity**	308 (42.4)	92 (41.4)	112 (41.2)	104 (44.6)	0.49	582 (44.9)	529 (45.3)	32 (48.5)	21 (33.3)	0.15
**Comorbidities**										
Hypertension	390 (53.7)	110 (49.6)	141 (51.8)	139 (59.7)	0.03	582 (44.9)	431 (36.9)	19 (28.8)	18 (28.6)	0.08
Dyslipidemia	355 (48.8)	91 (41.0)	141 (51.8)	123 (52.8)	0.01	702 (54.1)	635 (54.4)	35 (53.0)	32 (50.8)	0.56
Diabetes mellitus	83 (11.4)	24 (10.8)	29 (10.7)	30 (12.9)	0.48	76 (5.9)	70 (6.0)	2 (3.0)	4 (6.4)	0.75
**Body mass index** (kg/m^2^), median (IQR)	23.7 (22.0–25.9)	23.6 (21.9–26.0)	23.8 (22.0–25.6)	23.8 (22.0–25.8)	0.97	22.5 (20.5–24.7)	22.5 (20.5–24.6)	22.6 (20.8–25.1)	22.5 (20.3–25.8)	0.26
**Waist circumference** (cm), median (IQR)	84.5 (79.5–90.3)	84.1 (78.3–89.5)	84.2 (79.6–91.0)	85.2 (80.0–90.6)	0.06	81.1 (75.0–87.8)	81.2 (75.0–87.8)	80.3 (75.8–87.2)	81.0 (75.0–89.0)	0.53
**Employment**	486 (66.9)	154 (69.4)	200 (73.5)	132 (56.7)	0.004	684 (52.7)	601 (51.5)	43 (65.2)	40 (63.5)	0.01
**Education level^b^**					0.25					0.96
Low	102 (14.0)	34 (15.3)	28 (10.3)	40 (17.2)		188 (14.5)	169 (14.5)	6 (9.1)	13 (20.6)	
Middle	345 (47.5)	105 (47.3)	122 (44.9)	118 (50.6)		607 (46.8)	550 (47.1)	33 (50.0)	24 (38.1)	
High	280 (38.5)	83 (37.4)	122 (44.9)	75 (32.2)		502 (38.7)	449 (38.4)	27 (40.9)	26 (41.3)	

a For continuous variables, a linear trend test was used; for categorical variables, a Mantel–Haenszel χ^2^-test was used.

b Low, junior high school; Middle, high school; High, junior college, vocational technical school, or university. IQR: interquartile range.

**Figure 1:**
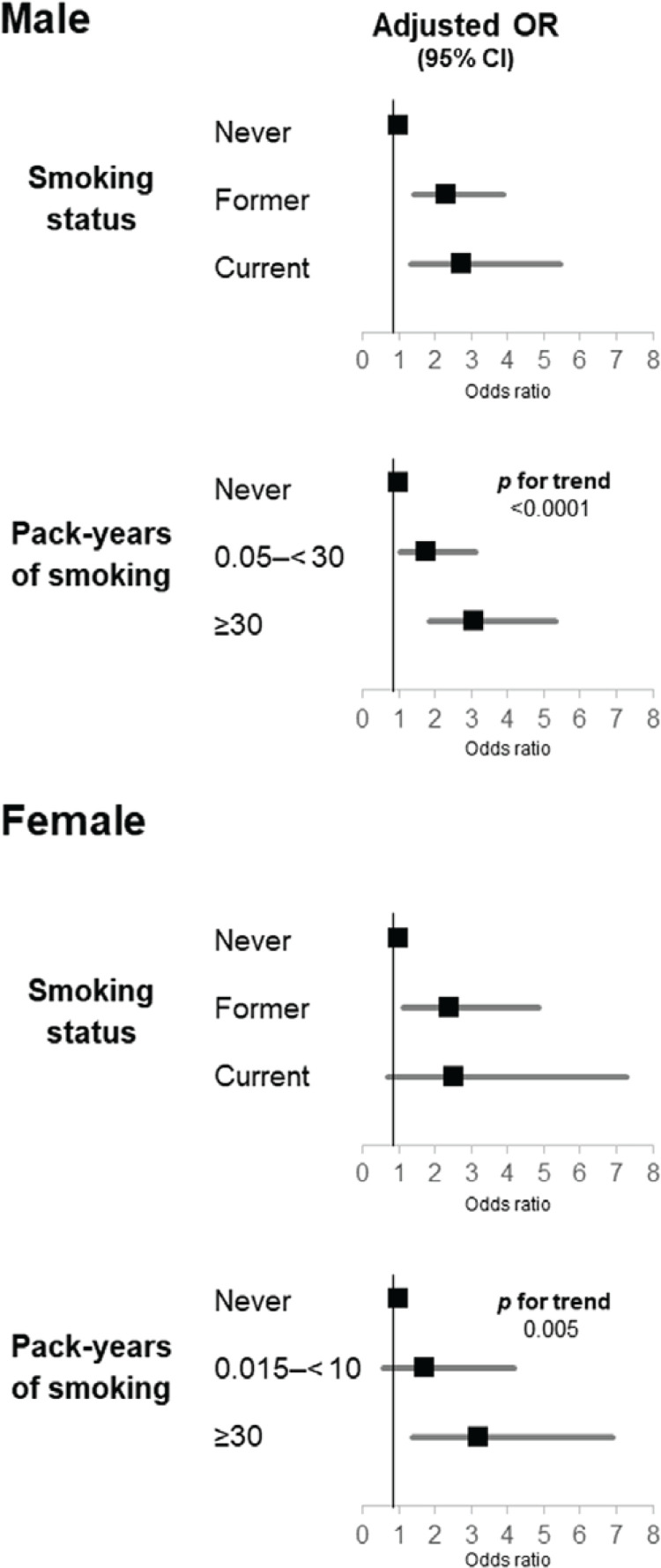
Multivariate-adjusted odds ratios for carotid wall thickening compared with having never smoked

Adjusted ORs and 95% CIs for carotid wall thickening and adjusted geometric means of maximum CIMT according to smoking status are given in Table 2. In men, compared with having never smoked, both former and current smoking were independently associated with a higher prevalence of carotid wall thickening; the multivariate-adjusted ORs were 2.32 (95% CI: 1.42–3.90) and 2.72 (95% CI: 1.35–5.47), respectively (Figure 1). Former, but not current, smoking was independently positively related to the maximum CIMT in comparison with having never smoked (p=0.02 and 0.14, respectively). Compared with having never smoked, 0.05 to <30 and ≥30 pack-years of smoking were independently positively associated with the prevalence of carotid wall thickening, demonstrating a clear exposure–response relationship; the multivariate-adjusted ORs were 1.77 (95% CI: 1.02–3.11) and 3.09 (95% CI: 1.84–5.34), respectively (p for trend <0.0001). A significant positive exposure–response relationship was found between pack-years of smoking and maximum CIMT. Among those who had never smoked and those with ≥30 pack-years of smoking, the multivariate-adjusted geometric means of maximum CIMT were 0.795 and 0.851, respectively (p for trend = 0.0003). In women, compared with having never smoked, only former smoking was independently associated with a higher prevalence of carotid wall thickening; the multivariate-adjusted OR was 2.41 (95% CI: 1.12–4.86). Compared with having never smoked, neither former nor current smoking was related to maximum CIMT (p=0.92 and 0.28, respectively). Compared with having never smoked, ≥10, but not 0.015 to <10, pack-years of smoking was independently positively associated with the prevalence of carotid wall thickening; the multivariate-adjusted OR was 3.22 (95% CI: 1.40–6.90) (p for trend = 0.005). There was no substantial relationship between pack-years of smoking and maximum CIMT. No significant interactions were found between smoking status or pack-years of smoking and sex affecting carotid wall thickening (p for interaction = 0.84 and 0.93, respectively). With regard to maximum CIMT, such interactions were not significant (p for interaction = 0.45 and 0.60, respectively).

**Table 2:** Adjusted odds ratios and 95% CIs for carotid wall thickening and adjusted geometric means of the maximum carotid intima-media thickness in relation to smoking status in 727 men and 1297 women, baseline data from the Aidai Cohort Study, Japan, 2015–2017

*Variables*	*Prevalence* *n/N (%)*	*Age-adjusted* *OR (95% CI)*	*Multivariate-adjusted* *OR (95% CI)^a^*	*Age-adjusted mean of maximum carotid intima-media thickness* *mm (95% CI)*	*Multivariate-adjusted mean of maximum carotid intima-media thickness* *mm (95% CI)^a^*
**Men**					
**Smoking status**					
Never	27/222 (12.2)	1	1	0.795 (0.774–0.816)	0.795 (0.774–0.816)
Former	92/395 (23.3)	2.21 (1.38–3.62)	2.32 (1.42–3.90)	0.829 (0.813–0.846)	0.829 (0.813–0.845)
Current	20/110 (18.2)	2.35 (1.20–4.55)	2.72 (1.35–5.47)	0.828 (0.797–0.860)	0.829 (0.798–0.861)
**Pack-years**					
None	27/222 (12.2)	1	1	0.795 (0.774–0.816)	0.795 (0.774–0.816)
0.05 to <30	46/272 (16.9)	1.72 (1.02–2.96)	1.77 (1.02–3.11)	0.811 (0.791–0.831)	0.810 (0.791–0.830)
≥30	66/233 (28.3)	2.78 (1.69–4.67)	3.09 (1.84–5.34)	0.851 (0.829–0.873)	0.851 (0.830–0.873)
p for trend		<0.0001	<0.0001	0.0004	0.0003
**Women**					
**Smoking status**					
Never	109/1168 (9.3)	1	1	0.762 (0.754–0.770)	0.763 (0.755–0.771)
Former	12/93 (12.9)	2.52 (1.22–4.84)	2.41 (1.12–4.86)	0.775 (0.746–0.804)	0.768 (0.740–0.797)
Current	4/36 (11.1)	2.16 (0.60–6.05)	2.54 (0.70–7.27)	0.795 (0.748–0.844)	0.797 (0.751–0.845)
**Pack-years**					
None	109/1168 (9.3)	1	1	0.762 (0.754–0.770)	0.763 (0.755–0.771)
0.015 to <10	6/66 (9.1)	1.82 (0.67–4.21)	1.70 (0.59–4.19)	0.763 (0.730–0.797)	0.758 (0.725–0.792)
≥10	10/63 (15.9)	3.03 (1.35–6.28)	3.22 (1.40–6.90)	0.799 (0.763–0.835)	0.796 (0.761–0.832)
p for trend		0.005	0.005	0.08	0.14

OR: odds ratio.

a Adjusted for age, alcohol consumption, leisure time physical activity, hypertension, dyslipidemia, diabetes mellitus, body mass index, waist circumference, employment, and education level.

The results for SHS exposure in the sample of 222 men and 1168 women who had never smoked are given in Table 3. In men, SHS exposure at home, pack-years of SHS exposure at home, SHS exposure at work, or years of SHS exposure at work was not associated with carotid wall thickening or maximum CIMT. In women, compared with never SHS exposure at home, current, but not former, SHS exposure at home was independently related to a lower prevalence of carotid wall thickening; however, no significant association was observed between SHS exposure at home and maximum CIMT. There were no relationships between pack-years of SHS exposure at home, SHS exposure at work, or years of SHS exposure at work and the two outcomes under study. There were no interactions between any of the SHS exposure categories and sex that affected the two outcomes under study (p for interaction = 0.26–0.99).

**Table 3:** Adjusted odds ratios and 95% CIs for carotid wall thickening and adjusted geometric means of the maximum carotid intima-media thickness in relation to secondhand smoke exposure in 222 men and 1168 women who had never smoked, baseline data from the Aidai Cohort Study, Japan, 2015–2017

*Variables*	*Prevalence* *n/N (%)*	*Age-adjusted* *OR (95% CI)*	*Multivariate-adjusted* *OR (95% CI)^a^*	*Age-adjusted mean of maximum carotid intima-media thickness* *mm (95% CI)*	*Multivariate-adjusted mean of maximum carotid intima-media thickness* *mm (95% CI)^a^*
**Men**					
**Secondhand smoke exposure at home**					
Never	20/165 (12.1)	1	1	0.789 (0.766–0.813)	0.785 (0.762–0.809)
Former	7/52 (13.5)	1.41 (0.51–3.56)	1.92 (0.63–5.52)	0.811 (0.768–0.856)	0.822 (0.778–0.869)
Current	0/5 (0)	Not calculable	Not calculable	0.788 (0.663–0.936)	0.787 (0.662–0.937)
**Pack-years of secondhand smoke exposure at home**					
None	20/165 (12.1)	1	1	0.789 (0.766–0.813)	0.785 (0.762–0.809)
0 to <10	3/28 (10.7)	1.17 (0.26–3.99)	1.47 (0.30–5.64)	0.805 (0.748–0.866)	0.815 (0.756–0.878)
≥10	4/29 (13.8)	1.37 (0.36–4.23)	2.03 (0.50–7.19)	0.813 (0.756–0.873)	0.824 (0.766–0.886)
p for trend		0.59	0.28	0.41	0.18
**Secondhand smoke exposure at work**					
Never	17/107 (15.9)	1	1	0.796 (0.767–0.827)	0.793 (0.763–0.824)
Former	8/92 (8.7)	0.52 (0.20–1.26)	0.49 (0.16–1.41)	0.791 (0.760–0.823)	0.794 (0.761–0.828)
Current	2/23 (8.7)	0.92 (0.13–3.97)	0.78 (0.10–4.05)	0.796 (0.733–0.864)	0.801 (0.736–0.871)
**Years of secondhand smoke exposure at work**					
None	17/107 (15.9)	1	1	0.796 (0.767–0.826)	0.793 (0.763–0.824)
1 to <23	4/60 (6.7)	0.56 (0.15–1.70)	0.46 (0.11–1.59)	0.799 (0.759–0.842)	0.803 (0.762–0.845)
≥23	6/55 (10.9)	0.57 (0.19–1.51)	0.62 (0.18–1.91)	0.784 (0.744–0.827)	0.787 (0.745–0.831)
p for trend		0.23	0.33	0.69	0.91
**Women**					
**Secondhand smoke exposure at home**					
Never	48/447 (10.7)	1	1	0.764 (0.751–0.777)	0.764 (0.752–0.777)
Former	55/508 (10.8)	1.10 (0.72–1.69)	1.08 (0.69–1.68)	0.771 (0.759–0.783)	0.771 (0.759–0.783)
Current	6/213 (2.8)	0.37 (0.14–0.84)	0.38 (0.14–0.87)	0.758 (0.739–0.776)	0.757 (0.739–0.776)
**Pack-years of secondhand smoke exposure at home**					
None	48/447 (10.7)	1	1	0.764 (0.751–0.777)	0.764 (0.752–0.777)
0 to <15	26/354 (7.3)	0.93 (0.55–1.56)	0.96 (0.55–1.64)	0.762 (0.747–0.776)	0.763 (0.749–0.778)
≥15	35/367 (9.5)	0.92 (0.56–1.47)	0.87 (0.53–1.42)	0.772 (0.758–0.787)	0.770 (0.757–0.784)
p for trend		0.71	0.58	0.41	0.56
**Secondhand smoke exposure at work**					
Never	70/657 (10.7)	1	1	0.765 (0.754–0.776)	0.765 (0.754–0.775)
Former	36/430 (8.4)	0.97 (0.62–1.50)	0.97 (0.61–1.55)	0.767 (0.754–0.780)	0.768 (0.755–0.781)
Current	3/81 (3.7)	1.15 (0.26–3.47)	1.26 (0.28–4.06)	0.768 (0.737–0.800)	0.765 (0.735–0.796)
**Years of secondhand smoke exposure at work**					
None	70/657 (10.7)	1	1	0.765 (0.754–0.775)	0.765 (0.754–0.775)
1 to <11	23/286 (8.0)	1.22 (0.71–2.04)	1.23 (0.69–2.12)	0.774 (0.757–0.790)	0.773 (0.756–0.789)
≥11	16/225 (7.1)	0.77 (0.42–1.35)	0.79 (0.42–1.41)	0.760 (0.742–0.778)	0.761 (0.744–0.779)
p for trend		0.55	0.60	0.84	0.93

OR: odds ratio.

a Adjusted for age, alcohol consumption, leisure time physical activity, hypertension, dyslipidemia, diabetes mellitus, body mass index, waist circumference, employment, and education level.

## DISCUSSION

The present cross-sectional study found that active smoking and pack-years of smoking were independently positively related to carotid wall thickening regardless of sex although the association with current smoking in women was not statistically significant. Independent positive relationships were shown between former smoking and pack-years of smoking and maximum CIMT only in men. In both sexes, we found no associations between SHS exposure at home or work and carotid wall thickening or maximum CIMT, whereas in women, current SHS exposure at home was significantly inversely related to carotid wall thickening.

A US cross-sectional study of 182 men and 136 women aged 33–42 years found a significant positive relationship between pack-years of smoking and mean CIMT in men; however, no such positive relationship was observed in women^15^. In a French cross-sectional study of 194 men and 330 women aged 17–65 years with no risk factors other than smoking, in men, current, but not former, smoking was associated with increased mean CIMT and a positive correlation was observed between pack-years of smoking and mean CIMT, while in women, such positive associations were not shown^14^. The Study of Women’s Health Across the Nation that included 1307 participants free of clinical CVD with a mean age of 60 years showed no association between smoking status and mean CIMT assessed on average 13.7 years after the baseline visit^26^. Our results are in partial agreement with these findings.

A cross-sectional study of 5032 non-smoking US adults aged 45–84 years without prior CVD found no association of SHS exposure in the past year with CIMT^7^. A US cohort study of 415 non-smoking adults revealed that SHS exposure in childhood only and SHS exposure in both childhood and adulthood, but not SHS exposure in adulthood only, were significantly associated with increased CIMT^8^. Our results are in partial agreement with these findings. In a cohort study using data from the Cardiovascular Risk in Young Finns Study (n=2401) and the Childhood Determinants of Adult Health (n=1375), adults who had been exposed to both parents smoking in childhood or adolescence had greater CIMT in adulthood than those who had not been exposed^4^. In the Atherosclerosis Risk in Communities Study, SHS exposure was significantly associated with a 20% increase in CIMT during the 3-year follow-up^27^. Our results were at variance with this finding.

Tobacco smoke contains numerous harmful substances, such as nicotine, carbon monoxide, and free radicals. These substances can enhance catecholamine secretion, increase erythrocyte and oxidized LDL levels, and induce inflammation, among other effects. These factors are believed to contribute to the onset and progression of atherosclerosis and CVD^28,29^. The observed lack of associations with SHS exposure may be attributed to lower levels of SHS exposure, given that levels of SHS exposure in the abovementioned Atherosclerosis Risk in Communities Study conducted in the US between 1987 and 1989^27^ were higher than those in the present study.

### Strengths and limitations

A number of methodological advantages were identified in the present study: participants had similar regions of residence, an automatic on-screen carotid ultrasound system was used, and several confounders were controlled for.

The present results should be interpreted in the context of its limitations. The cross-sectional nature of the current study precludes its use for proving causality. With regard to selection bias, study subjects were recruited from health checkup participants or through alternative recruitment procedures. The percentage participation was likely very low, although it could not be calculated because an accurate total of eligible study participants was not available. Thus, the current study population did not represent the general population of Japan, and our findings may not be generalized. For instance, the participants in the current study were more educated compared with the general population. According to the 2010 population census in Ehime Prefecture, the percentage of individuals between 60 and 69 years with a low, middle, high, or undisclosed educational level were 28.2%, 48.6%, 19.0%, and 4.2% in men, and 26.7%, 56.4%, 12.9%, and 4.0% in women, respectively^30^. The corresponding percentages for study participants in the same age range were 13.2%, 52.7%, 34.1%, and 0.0% in men, and 16.4%, 51.8%, 31.9%, and 0.0% in women, respectively. We were not able to assess differences between participants in the AICOS and non-participants because information on personal characteristics of non-participants was not available.

In the current study, smoking and SHS exposure were evaluated on the basis of participants’ questionnaire responses; no information on objective measures such as cotinine levels in saliva, serum, or hair was available. Participants may have intentionally underreported smoking activities to provide answers they deemed socially acceptable. Also, the number of cigarettes smoked in relation to SHS exposure at work were not available. Participants likely had the same probability of being misclassified in relation to their smoking and SHS exposure regardless of whether they had carotid wall thickening, and this type of non-differential exposure misclassification would lead to an underestimation of the strength of the association between exposure and outcome. That is to say, it would dilute the effect of the exposure.

An inherent weakness of the carotid ultrasound system employed here is that it cannot be used to quantify the amount of plaque shown in images. Although CIMT and plaque are distinct in phenotype and both denote elevated vascular risk, CIMT, even in the absence of plaque, is an important indicator of heightened risk for vascular incidents and reliably signals the likelihood of plaque formation^31^. In addition, while the accuracy of CIMT measurement using an automated onscreen carotid ultrasonography device has been validated in previous studies^19,20^, we did not verify the inter- and intra-observer measurement accuracy for CIMT within our study. Furthermore, in the present study, the maximum CIMT was defined solely based on the common carotid artery. Typically, the maximum CIMT also encompasses evaluations of the internal carotid artery and carotid bulb. However, the reproducibility of measuring CIMT in the internal carotid artery, carotid bulb, and common carotid artery is lower compared to measuring only the common carotid artery site^32^. One reason is that in Japanese individuals, compared to Western populations, the carotid bifurcation often lies higher than the angle of the mandible^33^, making evaluations of the internal carotid artery and carotid bulb more challenging. Therefore, considering the potential issues with reproducibility, we decided to assess IMT based only on the common carotid artery. The observed null associations of active smoking and SHS exposure with the outcomes under study might be ascribed to insufficient statistical power. In particular, the associations with SHS exposure in men were assessed among only 222 participants. Residual confounding effects could not be ruled out although we adjusted for multiple confounders.

## CONCLUSIONS

The present cross-sectional study in Japan showed that active smoking (except for current smoking in women) and pack-years of smoking were independently related to a higher prevalence of carotid wall thickening in both sexes, while independent positive associations between former smoking and pack-years of smoking and maximum CIMT were observed only in men. Given the limited evidence of the association between smoking and subclinical atherosclerosis in Asian populations, further evidence is necessary to better understand the present findings.

## Data Availability

The data supporting this research cannot be made available for privacy or other reasons.
